# Contradictory roles of lipid metabolism in immune response within the tumor microenvironment

**DOI:** 10.1186/s13045-021-01200-4

**Published:** 2021-11-06

**Authors:** Weina Yu, Qingyang Lei, Li Yang, Guohui Qin, Shasha Liu, Dan Wang, Yu Ping, Yi Zhang

**Affiliations:** 1 Biotherapy Center and Cancer Center, The First Affiliated Hospital of Zhengzhou University, Zhengzhou, 450052 Henan People’s Republic of China; 2 Henan Key Laboratory for Tumor Immunology and Biotherapy, Zhengzhou, 450052 Henan China; 3 School of Life Sciences, Zhengzhou University, Zhengzhou, 450001 People’s Republic of China; 4 State Key Laboratory of Esophageal Cancer Prevention & Treatment, Zhengzhou University, Zhengzhou, 450052 People’s Republic of China

**Keywords:** Lipid metabolism, Malignant tumor, Immune response, Tumor microenvironment, Immunotherapy

## Abstract

Complex interactions between the immune system and tumor cells exist throughout the initiation and development of cancer. Although the immune system eliminates malignantly transformed cells in the early stage, surviving tumor cells evade host immune defense through various methods and even reprogram the anti-tumor immune response to a pro-tumor phenotype to obtain unlimited growth and metastasis. The high proliferation rate of tumor cells increases the demand for local nutrients and oxygen. Poorly organized vessels can barely satisfy this requirement, which results in an acidic, hypoxic, and glucose-deficient tumor microenvironment. As a result, lipids in the tumor microenvironment are activated and utilized as a primary source of energy and critical regulators in both tumor cells and related immune cells. However, the exact role of lipid metabolism reprogramming in tumor immune response remains unclear. A comprehensive understanding of lipid metabolism dysfunction in the tumor microenvironment and its dual effects on the immune response is critical for mapping the detailed landscape of tumor immunology and developing specific treatments for cancer patients. In this review, we have focused on the dysregulation of lipid metabolism in the tumor microenvironment and have discussed its contradictory roles in the tumor immune response. In addition, we have summarized the current therapeutic strategies targeting lipid metabolism in tumor immunotherapy. This review provides a comprehensive summary of lipid metabolism in the tumor immune response.

## Background

Discriminating between transformed cells and normal cells by recognizing unique antigens and combating malignant tumors with humoral and cell-mediated immune responses are critical functions of the immune system [1]. Many intrinsic molecules change in the early stages of cancer initiation, resulting in a distinct immune response pattern compared to the normal. Some factors produced by tumor cells, such as double-stranded DNA, may trigger the host immune system to generate an effective immune response and reduce the incidence of tumors [2]. This anti-tumor immune response also termed “immune surveillance,” generally leads to the elimination and control of cancer cells throughout the development of malignant tumors, especially in the early stage [3]. However, the immune system cannot always detect and kill all cancer cells. These surviving tumor cells remold local immune cells and stromal cells to build themselves a survivable environment termed “tumor microenvironment (TME)” and to escape from immune surveillance and elimination [4]. Many factors in the TME drive immune cells to a pro-tumor phenotype and facilitate the proliferation and metastasis of tumor cells. These immune responses are based on tumor-infiltrating immune cells, including T cells, macrophages, dendritic cells (DCs), neutrophils (Ns), natural killer (NK) cells, and myeloid-derived suppressive cells (MDSCs). T cells are the critical participants in the entire process of tumor development. CD8^+^ T cells can differentiate into cytotoxic T lymphocytes (CTL) and directly eliminate tumor cells in a major histocompatibility complex (MHC)-dependent manner [5]. CD4^+^ T cells include pro-inflammatory T-helper 1(Th1) cells, immunosuppressive T-helper 2 (Th2) cells, ambiguous T helper 17 (Th17) cells, and immune regulatory T cells (Tregs). Each of these has distinctive effects on tumor immunology [6]. Tumor-infiltrating macrophages are classified as classically activated (M1) macrophages and alternatively activated (M2) macrophages. M1 macrophages are prone to participate in the anti-tumor immune response by producing pro-inflammatory cytokines and inducible nitric oxide synthase (iNOS). In contrast, M2 macrophages exhibit an anti-inflammatory phenotype and secrete many pro-tumor factors, such as arginase 1 (ARG1) [7]. Although Ns are important immune cells that participate in the inflammatory process, they affect the TME in two distinct ways. The N1 subtype secretes pro-inflammatory cytokines and elicits a cytotoxic immune response, while the N2 subtype is supposed to function as immunosuppressive cells [8]. MDSCs are a group of immature myeloid cells that play critical roles in tumor development, metastasis, and therapy resistance. Based on their origin, tissue localization, and mechanism of immune suppression, MDSCs can be divided into monocytic MDSCs and polymorphonuclear MDSCs. They exhibit immunosuppressive capability by inhibiting other immune cells in a context-dependent manner [9, 10]. Besides, NK cells and DCs are thought to participate in potent anti-tumor immune response, but various immunosuppressive factors tend to restrain their abilities and drive them into pro-tumor phenotypes [11–13]. Except for immune cells, cancer-associated fibroblasts (CAFs) represent a dominant component of the tumor stroma and contribute significantly to the formation of the TME. CAFs can facilitate the malignant phenotype of tumor cells, reinforce therapy resistance, and modulate other tumor-infiltrating immune cells by depositing and remodeling the extracellular matrix, and secreting cytokines or direct cell–cell contact [14].

**Fig. 1:**
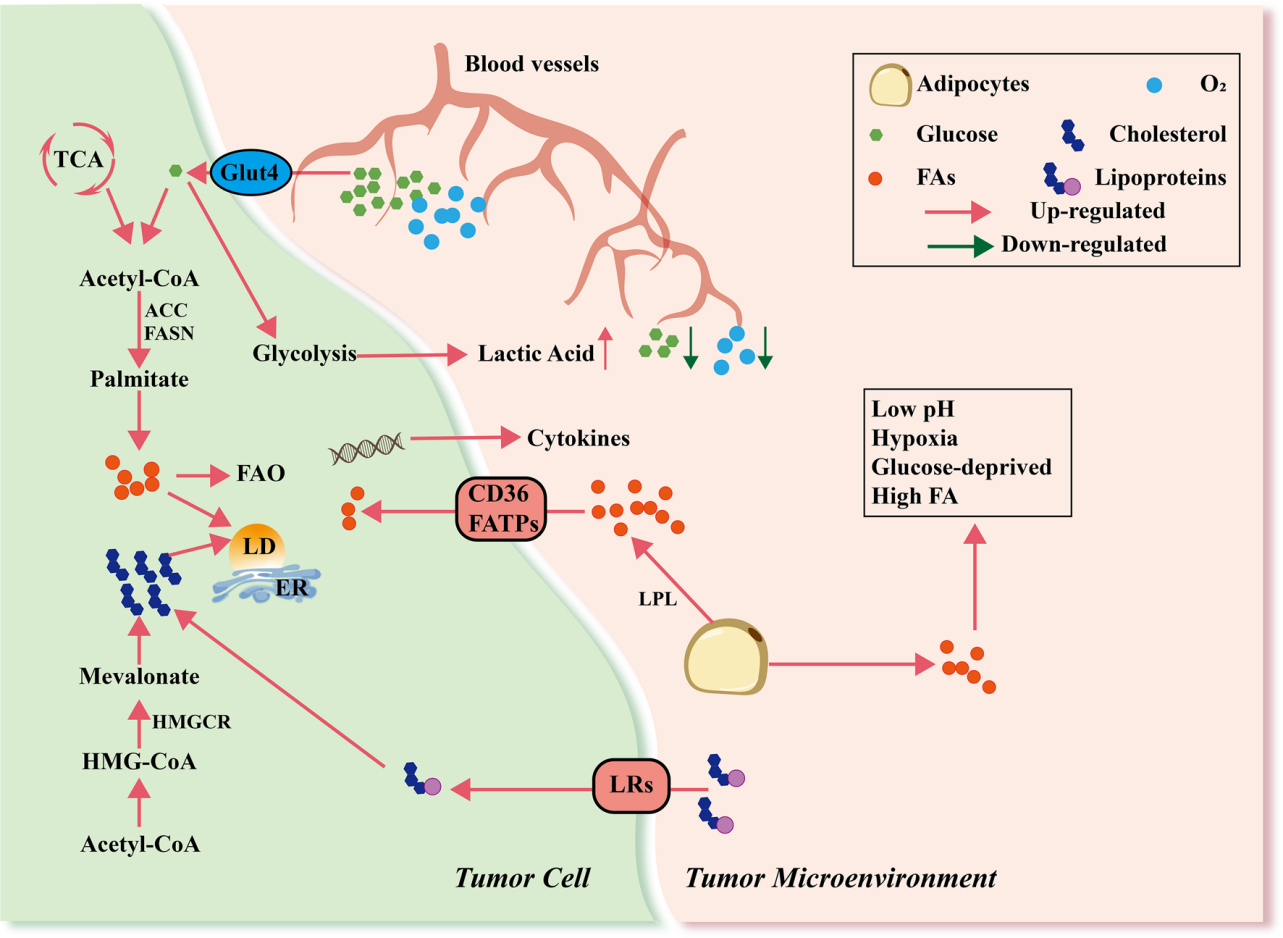
Metabolic reprogramming in the tumor microenvironment. In the tumor microenvironment, most of the glucose and oxygen transported by disorganized blood vessels are taken up by tumor cells, leading to hypoxia and a glucose-deprived microenvironment. Activated glycolysis in tumor cells generates increased lactic acid but still does not satisfy energy needs. As a result, LPL from tumor cells and other stromal cells activates adipocytes and induces lipolysis of stored triglycerides and secretion of FAs, which are transported into cells through CD36 or FATPs. In addition, tumor cells can generate FAs via de novo synthesis pathway using acetyl-CoA from the catabolism of glucose. ACC and FASN participate in this process. These FAs then participate in FAO or other signaling pathways to produce many immunosuppressive factors or generate LDs. In addition, lipoproteins in the TME are transported via lipoprotein receptors (LRs) and catabolize to cholesterol in cells. Tumor cells can also generate cholesterol via the mevalonate pathway. Dysregulated lipid metabolism in tumor cells promotes the formation of acidic, hypoxic, glucose-deprived, and lipid-rich immunosuppressive microenvironments

Energy and metabolites are indespensible in cell survival. Malignant tumor cells require extensive energy and raw materials to support their uncontrolled proliferation and sustenance of daughter cells. Although angiogenesis in the TME is increased, it can barely meet the glucose and oxygen demands of the expansion and dissemination of cancer cells, consequently leading to a glucose-deficient and hypoxic microenvironment. This forces malignant tumor cells to adjust their metabolic profiles to sustain activated proliferation. In the 1920s, Otto Warburg et al. first proposed that tumor tissues metabolize approximately ten-fold more glucose to lactic acid than normal tissues in a given time regardless of the oxygen content in the TME, which is known as the Warburg effect [15]. Since then, metabolic reprogramming has become a promising field in the study of malignant tumors (Fig. 1). In recent years, the effects of lipids on tumor development have drawn increasing attention. In addition to being used as an alternative source to cover for energy shortages [16], lipids also participate in the synthesis of biological membranes, provide substrates for biomass production, and activate complex signaling pathways related to cancer cell growth and migration [17]. Cancer cells exhibit increased lipid and cholesterol avidity, such as elevated intake of exogenous lipids and lipoproteins and over-activated de novo synthesis, which directly contributes to malignant transformation and progression of tumor cells and abnormal lipid accumulation in the TME [18] (Fig. 1). Lipid metabolism dysfunction in the TME has far-reaching effects on tumor-associated immune responses. According to their effects on tumor cells, immune responses can be divided into pro-tumor immune response and anti-tumor immune response, both of which involve various immune cells such as T cells, macrophages, DCs, and MDSCs. Due to the complex composition of lipids and underlying mechanisms, the same type of immune cells may respond very differently to changes in lipid metabolism and lead to ambiguous conclusions. For example, excessive amounts of free fatty acids (FFAs) inhibit the CTL-mediated killing of tumor cells, which can be recovered by decreasing FFA levels [19]. However, in a harsh TME, tissue-resident memory (Trm) cells are prone to take in FAs via CD36 to support the anti-tumor response and their long-term survival [20]. Therefore, determining the exact underlying lipid metabolic mechanisms of immune cells is vital to the understanding of immunosuppressive phenomena and the development of effective combined therapies.

In this review, we have discussed the complex effects of lipid metabolism on the tumor-associated immune response from two perspectives. In addition, we have explored current strategies and molecular targets aimed at the lipid metabolism in the TME, while describing the limitations and future directions.

## Basic concepts of lipids metabolism

Lipids are divided into three general categories based on their structure: (a) simple lipids containing long-chain FAs and their esterification products, such as triacylglycerol (TAG), (b) derived lipids like steroids, fat-soluble vitamins, and carotenoids, and (c) compound lipids containing various functional groups, such as phospholipids, glycolipids, and lipoproteins (LP).

### Fatty acid

Exogenous FAs are mainly derived from TAGs, which are digested primarily in the upper jejunum by the catalytic activity of pancreatic lipase [21]. These FFAs are then absorbed from the intestinal lumen via CD36 or fatty acid transporter protein (FATP) into the enterocytes and then re-assembled into TAG by monoacylglycerol acyltransferase and diacylglycerol acyltransferase enzymes [22, 23]. In order to be transported in the blood, TAGs usually bind to LPs and form chylomicrons, very low-density LPs (VLDL), low-density LPs (LDL), and high-density LPs (HDL) [24]. LPs can be hydrolyzed by lipoprotein lipase (LPL) or transported into cells via corresponding receptors to generate FAs and proteins [25]. De novo synthesis is another primary source of FAs. ATP-citrate lyase (ACLY) catalyzes the conversion of citrate to acetyl-coenzyme A (acetyl-CoA), which is then converted to malonyl-CoA by the enzyme acetyl-CoA carboxylase (ACC). Repeated condensation of acetyl-CoA and malonyl-CoA by fatty acid synthase (FASN) generates palmitic acid, which turns into many other types of FAs via further elongation and desaturation [26] (Table 1).

**Table 1:** Basic concepts of primary lipids

Source	Key enzymes	Category	Functions	Regulators	References
Exogenous	CD36 FATP MAGT DAGT LDLR	Fatty acid	FAO	ACS CPT1	[22, 23, 26-30]
Endogenous	ACLY ACC FASN		Regulate signaling pathways and gene transcription	PPARs SREBP-1	
			Physical processes and component formation		
Exogenous	LDLR VLDLR		CE synthesis	ACAT	[31-34, 37-39]
Endogenous	HMGCR	Cholesterol	Cholesterol efflux	ABCA1 ABCG1	
			Regulate signaling pathways and gene transcription	LXRs	
			Physical process and component formation		
Endogenous		Lipid droplet	Store energy	ATGL HSL MAGL LAL	[41-44]
			Keep lipid metabolism balance		

In cells, FAs primarily act as an energy source through fatty acid oxidation (FAO). Fatty acyl CoA synthetase (ACS) and carnitine palmitoyltransferase I (CPT1) are critical enzymes in the generation of fatty acyl-CoA and fatty acylcarnitine [27]. After shuttling into mitochondria, acylcarnitine transforms into acyl-CoA and enters the tricarboxylic acid cycle to generate adenosine triphosphate (ATP) and nicotinamide adenine dinucleotide phosphate (NADP), which counteracts oxidative stress [28]. Furthermore, FAs regulate cellular signaling pathways and gene transcription by binding to and activating the nuclear receptor family of transcription factors, such as peroxisome proliferator-activated receptors (PPARs), which control the expressions of genes involved in lipid and energy homeostasis and inflammation. PPAR-α directly upregulates the expression of CPT1α and PPAR-δ regulates lipids delivery and oxidation [29]. In addition, sterol regulatory element-binding protein 1 (SREBP-1) transcription factors affect the expressions of genes involved in the lipid metabolism such as ACLY, ACC, and FASN [30] (Table 1).

### Cholesterol

Cholesterol is an indispensable component of lipid metabolism. Its absorption is accompanied by FAs through CD36 and Niemann–Pick C1 like 1, and then they are re-assembled into chylomicron [31]. Similar to FAs, cholesterol is packaged into very low-density LPs and low-density LPs in the liver for transportation in the circulation. After entering cells via corresponding receptors, these LPs are hydrolyzed to free cholesterol by cholesterol ester hydrolase in the lysosome. However, diet-derived cholesterol is not able to satisfy physiological requirements, and its de novo synthesis via the mevalonate pathway is critical [32]. 3-hydroxy-3-methylglutaryl coenzyme A reductase (HMGCR) is the rate-limiting enzyme in this process, mediates the conversion of 3-hydroxy-3-methylglutaryl coenzyme A to mevalonic acid, which then enters the downstream multi-step synthesis [33] (Table 1).

To avoid the cytotoxicity of extensive accumulation of free intercellular cholesterol, acyl-coenzyme A-cholesterol acyltransferase (ACAT) transforms cholesterol into cholesterol ester (CE) and stores it in lipid droplets (LDs) [34]. ACAT2 deficiency dramatically downregulates the absorption rate of cholesterol, activates SREBP, and enhances the transcription of many regulatory proteins, such as HMGCR and lipoprotein receptor (LDLR), to increase cholesterol uptake and synthesis [35, 36]. In addition, ATP-binding cassette (ABC) proteins mediate cholesterol efflux in cells, especially in macrophages [37]. Liver X receptors (LXR) promote lipogenesis and transcription of ABCA1 and ABCG1 [38]. Many physical processes depend on cholesterol, such as the formation of the plasma membrane, caveolae, and lipid rafts, biosynthesis of some molecules, such as dolichol and coenzyme Q, and structural backbone for metabolites [39]. However, an overabundance of cholesterol reduces membrane fluidity, destroys lipid raft signaling, generates damaging oxidative molecules, and ultimately results in cell death [40] (Table 1).

### Lipid droplets

LDs are important subcellular organelles that regulate cellular lipid metabolism by maintaining the balance of lipid storage and breakdown and protecting cells from potentially toxic lipids [41]. LD contains a lipid core consisting of TAG, sterol esters, and FFAs, which is surrounded by a monolayer consisting of phospholipids and cholesterol [42]. Various enzymes and proteins involved in lipid metabolism, such as adipose triglyceride lipase (ATGL), hormone-sensitive lipase (HSL), monoacylglycerol lipase (MAGL), and lysosomal acid lipase (LAL) are located on the surface of LDs and participate in the regulation of lipid metabolism [43]. PPAR agonists, glucocorticoids, and fasting elevate ATGL expression, whereas insulin and food intake have the opposite effect [44] (Table 1).

These complex lipid components and underlying metabolic processes are abnormally regulated in the TME, and thus exert a significant impact on tumor immune responses.

## Anti-tumor effects of lipids in tumor immune response

Nutrient limitation caused by an insufficient vascular exchange in the TME can result in intense competition between immune cells and rapidly proliferating cancer cells, which modifies anti-tumor immune defense [45]. Immune cells and immunological mediators are activated to participate in the host defense against malignant tumors. CTLs, NK cells, macrophages, and DCs are the predominant effector cells in tumor detection and elimination. Although tumor cells build a harsh TME, these effector immune cells still find new ways to survive and use lipids as fuel to support anti-tumor immune responses.

### The role of lipids in antigen-specific anti-tumor immune response

**Fig. 2:**
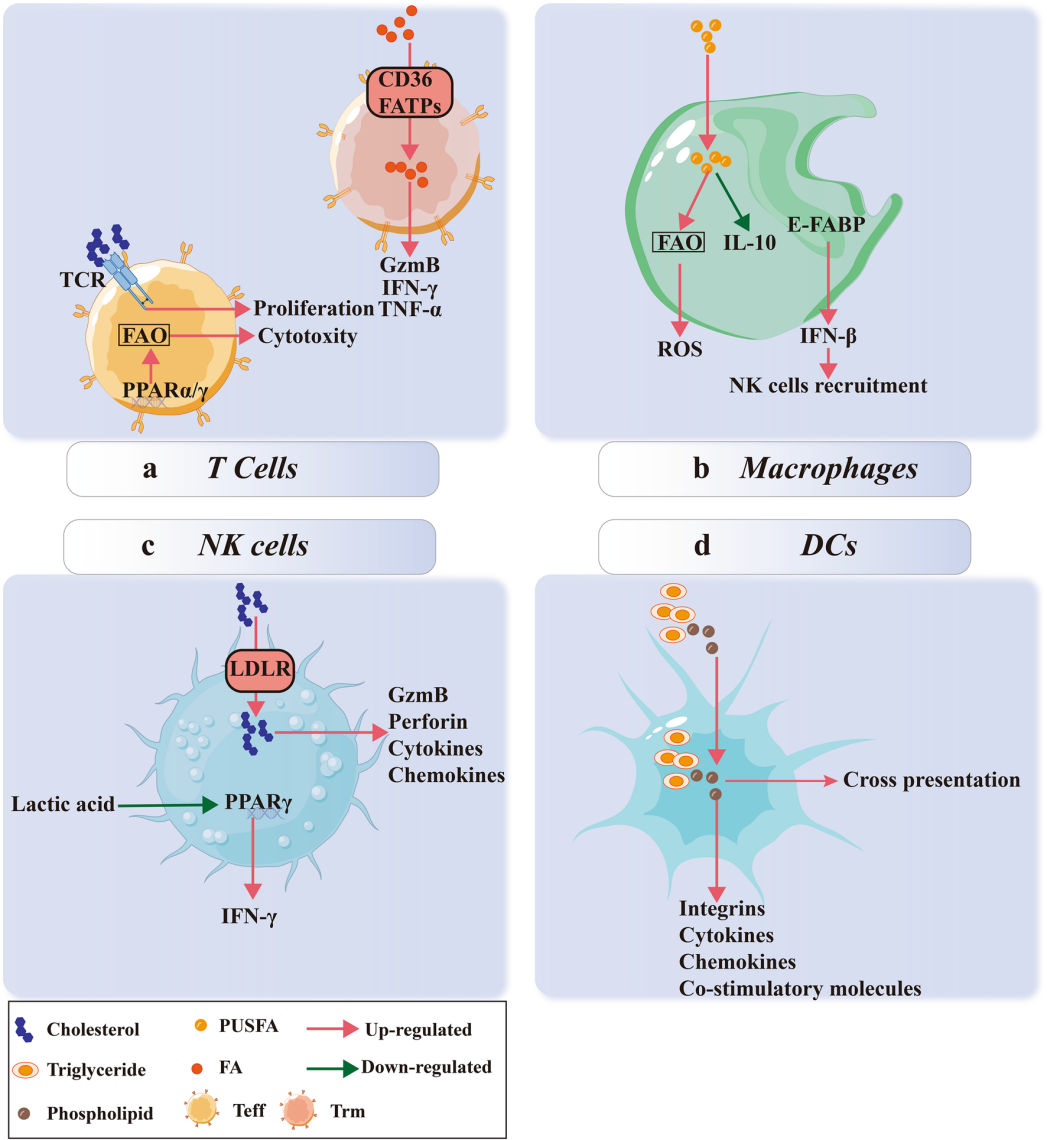
Lipid metabolism in anti-tumor immune response. **a** Trms take in FAs from the TME via CD36 and FATPs to generate anti-tumor cytokines like GzmB, IFN-γ, and TNF-α. Moreover, cholesterol helps the formation of TCRs on Teffs and stimulates their proliferation and cytotoxicity. PPAR-α/γ also enhances the anti-tumor ability of Teffs through activating FAO; **b** FAs in the TME enhance FAO in macrophages, which upregulate ROS production, downregulate IL-10 secretion, and eliminate tumor cells. Elevated E-FABPs in macrophages promote IFN-β expression and mediates the recruitment of NK cells to kill tumor cells; **c** Cholesterol taken in via LDLR stimulates the expression of effector markers (GzmB and perforin), cytokines, and chemokines. Activated PPAR-γ in NK cells promotes the secretion of IFN-γ, which is suppressed by excessive lactic acid in the TME; **d** triglyceride and phospholipid enhance the cross-presentation ability and cytokine secretion level of DCs to participate in the anti-tumor response

CD8^+^ T cells are regarded as the most critical executors of adaptive anti-tumor immunity. Tumor-specific MHC-restricted CTLs can lyse neoplastic cells without influencing normal cells [46]. As the essential components of T cells, intracellular FAs supply energy through FAO and phosphorylation. Increased FAs in the TME activates PPARα signaling in CD8^+^ T cells, facilitating their lipid metabolism and preserving effector functions [47]. Besides, promoting FA catabolism of tumor-infiltrating CD8^+^ T cells improves their anti-tumor activity. Activation of PPAR-γ, a critical ligand-inducible nuclear receptor in lipid metabolism, facilitates differentiation of naïve T cells into effector T cells (Teffs) by upregulating FAO and inhibiting apoptosis of Teffs. In addition, it increases the abundance and activity of effector/memory CD8^+^ T cells in the drained lymph nodes and tumor sites [48] (Fig. 2a). Depletion of lipids in CD8^+^ T cells remarkably impedes their proliferation and cytotoxicity, leading to functional exhaustion characterized by reduced interferon (IFN)-γ and high programmed cell death protein-1 (PD-1) expression [49, 50]. PD-1 signaling inhibits TCR- and CD28-mediated activation of the PI3K/AKT/mTOR pathway, which in turn promotes lipolysis and FAO [51].

It has been reported that the application of anti-PD-1 antibody suppresses the FAO level of T cells and results in cell death due to their over-activation [52]. These results suggest that combining FAO activators with an anti-PD-1 antibody could reverse repression of the FAO level and its suppressive effects on T cell differentiation. Bezafibrate, a pan-PPAR agonist, can increase FAO levels under PD-1 blockade, as well as the proliferation and anti-tumor ability of CD8^+^ T cells [48]. In contrast, knockout of PPAR-α impairs FA catabolism and anti-tumor activity of CD8^+^ T cells accompanied by PD-1 expression [53]. In addition to Teffs, tumor-infiltrating CD8^+^ Trm T cells also exhibit anti-tumor activity by releasing lytic granules, granzyme B (GzmB), IFN-γ, and tumor necrosis factor (TNF)-α [54, 55] (Fig. 2a). High intercellular FA levels mediated by CD36 help sustain effective function and long-term survival of CD8^+^ Trm cells and reverse the apoptosis induced by cancer cell-caused lipid-deprivation [20]. Furthermore, programmed death-ligand 1 (PD-L1) blockage increases the expression of fatty acid-binding protein (FABP) 4/5 and lipid uptake, prolonging the survival span of Trm cells both in vitro and in vivo [20]. These results imply a potential link between FA metabolism and the anti-tumor effects of T cells, especially with immune checkpoints. Enhancing FAO in CD8^+^ T cells strengthens their anti-tumor response, especially when combined with anti-PD-1 therapy. As a result, T cell-mediated anti-tumor immune response is at least partially enhanced by FA metabolism, which means that stimulating FA uptake and FAO in T cells could be a therapeutic method in malignant tumor treatment. Future clinical treatment of malignant tumors should combine therapy with the intervention of lipid metabolism in T cells.

To verify this hypothesis, researchers have explored the effects of FAs in chimeric antigen receptor (CAR) T cells, an effective option for relapsed or refractory malignant tumors [56]. 4-1BB CAR-T cells facilitates the outgrowth of CD8+ central memory T cells, which use FAs as the predominant energy source and extensively rely on FAO [57]. The inclusion of 4-1BB in CAR architecture leads to higher FAO, respiratory capacity, and growth rate than CD28 CAR-T cells, which are characterized by high glycolysis [57]. These results suggest that modification of FA metabolism in CAR-T cells could influence their survival, function, and anti-tumor immune response after injection, suggesting a promising direction for T cell therapy.

Cholesterol is another critical regulator of T cell-mediated anti-tumor immune response. Free cholesterol in T cell membranes is an important component of T cell receptors (TCRs) and T cell immunological synapses that directly regulate signaling pathways and effector functions [58] (Fig. 2a). Elevation of cholesterol levels by blocking cholesterol esterification with ACAT1/2 inhibitors facilitates the direct movement of TCR microclusters to the synapse center, accompanied by elevated numbers and anti-tumor activity of CD8^+^ T cells both in vitro and in vivo [59]. Meanwhile, the inhibition of ACAT1 strengthens the tumor-limiting activities of CD19-CAR-T cells by elevating the ratio of effector-to-target cells even under low infection rates and could rescue CD8^+^ T cell inhibition in chemo-immunotherapy [60, 61]. Moreover, natural killer T (NKT) cells, a cluster of CD1d restricted T cells that simultaneously possess the characteristics of adaptive and innate immune cells, mostly recognize lipid antigens and exert multiple immunoregulatory effects [62]. Thus, lipids can regulate the cytotoxicity of NKT cells. Excessive glycolysis in tumor cells results in an abundance of lactic acid in the TME, which reduces PPAR-γ expression levels in tumor-infiltrating invariant NKT cells (iNKT) and thereby diminishing lipid synthesis and IFN-γ production. The PPAR-γ activator reverses this phenomenon and strengthens the anti-tumor activity of iNKT [63] (Fig. 2c). Moreover, in vitro experiments showed that only water-soluble cholesterol, but not other types of lipids, could successfully restore IFN-γ production and enhance TCR signaling at the immunological synapse of iNKT cells. Inhibition of cholesterol synthesis by simvastatin significantly impaired this effect [63].

### Lipids promote the non-specific anti-tumor immune response

FAs and cholesterol work as fuels and structural components of tumor-infiltrating T cells and facilitate antigen-specific detection and elimination of tumor cells. Unlike NKT cells, the anti-tumor immune response induced by NK cells is non-specific. The primary anti-tumor mechanisms for NK cells include recognition and conjugation of effectors to targets, delivery of death signals, and disintegration of tumor cells [64]. Cholesterol enhances the anti-tumor activity of NK cells. A high-cholesterol diet elevates the amount of NK cells and upregulates the levels of activating receptors and effector proteins, such as GzmB and perforin, in NK cells [65]. LDLR mediates the accumulation of cholesterol in NK cells, stimulates immune-activating ability, and reduces cancer proliferation [65] (Fig. 2c). Specific LDLR overexpression in tumor-infiltrating NK cells might be an optimal method to mobilize NK cells toward an anti-tumor immune response. Thus, the accumulation of cholesterol in NK cells might be a powerful method to awaken their anti-tumor ability in an immunosuppressive TME and exert tumor-eliminating effects, alone as well as synergistically.

In the TME, macrophages and DCs play roles in antigen presentation and display anti-tumor effects mainly through antibody-dependent cytotoxicity and soluble cytotoxicity factors. Obesity has been reported to accumulate pro-inflammatory macrophages with increased levels of reactive oxygen species (ROS) levels [66] (Fig. 2b). However, this phenomenon is non-universal and is largely related to the FA subtype. For example, fish oil-fed mice possess a lower percentage of pro-tumor macrophages and interleukin (IL-10) expression and higher infiltrations of B cells and CD^+^ 8 T cells than coco butter-fed mice [67]. In addition, the enrichment of polyunsaturated fatty acids (PUSFAs), not saturated fatty acids (SFAs), exerts higher cytotoxicity on macrophages against tumor cells [68]. This gives rise to the hypothesis that some FAs might enhance the anti-tumor immune response of macrophages, but others do not. N-3 docosapentaenoic acid, an unsaturated fatty acid in fish oil, specifically increases the expression of FAO-related genes (*A-FABP*, *Cpt1b*, *PCX*, and *UCP2*) and apoptosis-related genes (*iNOS*, *RIPK3*, *caspase-8*, and *caspase-11*), that induce ROS-mediated macrophage apoptosis and reduce pro-tumor activity. High E-FABP-expressing macrophages have enhanced IFN-β production and recruit more tumoricidal immune cells, especially NK cells [69] (Fig. 2b). Moreover, enzyme-instructed self-assembly of phosphotyrosine-cholesterol reeducates pro-tumor macrophages to an anti-tumor phenotype by inducing ROS production, disrupting filaments in macrophages, and inhibiting ovarian cancer cell growth [70]. This suggests a new way of polarizing and even reeducating tumor-infiltrating macrophages into an anti-tumor phenotype by providing exogenous modified lipids.

Unlike other immune cells, the type of lipid (saturated vs. unsaturated), rather than the amount of lipid, is more critical for DCs to process antigens or release anti-tumor cytokines [71]. DCs with higher lipid contents tend to accumulate phospholipids and TAGs, but not cholesterol and cholesteryl esters. These DCs have higher levels of integrins, co-stimulatory molecules, glycoproteins, pro-inflammatory cytokines, and chemokines than the DCs with low lipid content (Fig. 2d). Moreover, in vivo experiments showed that higher lipid content rendered DCs with stronger cross-presentation ability, potently activated NK and NKT cells, and enhanced endogenous CTL. However, low lipid content was more likely to induce anergy [71]. These results have been confirmed in mice immunized with high lipid content DCs, which showed robust target lysis both in the spleen and liver and accelerated proliferation of OVA-restricted CD8^+^ Teffs, as well as delayed tumor development and smaller lesions [72]. Although researches about the lipids in DCs are still limited, it is seen that FAs may act as pro-inflammatory factors and enhance the anti-tumor activity of CTL in a DC-dependent manner, resulting in restrained tumor growth. However, whether this phenomenon is universally seen in other tissues and cancer types other than the liver needs to be investigated.

Highly proliferating tumor cells give rise to a glucose-deficient and lipid-enriched TME, which has been shown to facilitate their growth and dissemination and give rise to an immunosuppressive milieu. However, it needs to be mentioned that lipids can also function as indispensable fuels and metabolic components to facilitate the anti-tumor activity of immune cells. Current lipid metabolism-targeted therapies for malignant tumors are more devoted to simply impeding pan-lipid metabolism in the TME and achieve limited effects. As discussed above, specific activation of metabolic pathways in some immune cells could dramatically stimulate anti-tumor immune responses and facilitate anti-tumor therapy.

## Immunosuppressive effects of lipids in the tumor microenvironment

Although the immune system is devoted to surveilling and eliminating transformed cells, some cunning tumor cells can disguise themselves, leading to immune evasion. To build a suitable environment for growth, tumor cells remodel surrounding stromal cells and reeducate immune cells from an anti-tumor phenotype to bystanders or even pro-tumor phenotypes [4]. As mentioned above, intensive competition between tumor cells and immune cells for nutrient substances finally leads to a glucose-deficient and anoxic microenvironment, accompanied by excessive lipid accumulation and activated lipid metabolism. Notwithstanding the above tumor-limiting effects of immune cells caused by activated lipid metabolism, abnormal lipid status also suppresses the anti-tumor ability of immune cells and even reeducates them to the pro-tumor phenotype. This inconsistency may be due to differences in the cancer types or lipid species. Therefore, an in-depth discussion of lipid metabolism and the pro-tumor immune response is critical to reverse the immunosuppressive TME without disrupting the anti-tumor immune response.

### Accumulation of FAs damages the cytotoxicity of effector T cells

Recent studies have reported that naïve T cells rely more on mitochondrial respiration and FAO to minimize the cell-damaging effects of toxic metabolites. However, to sustain the high proliferation rate and activities, Teffs are prone to utilize glycolysis and aerobic respiration to satisfy their need for ATP [73]. This means that a lipid-enriched TME might suppress the anti-tumor effects of Teffs.

**Fig. 3:**
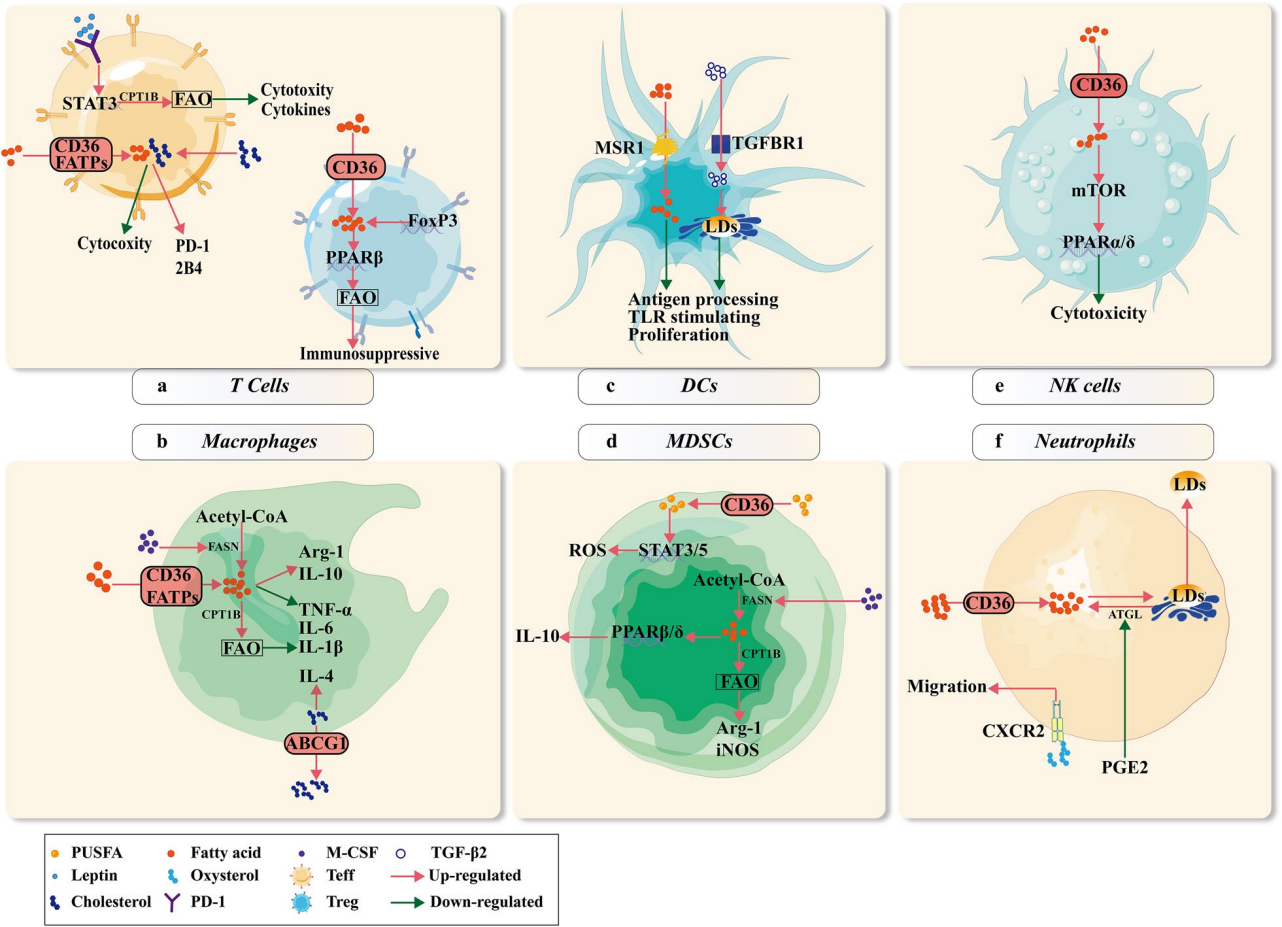
Lipid metabolism in pro-tumor immune responses. **a** FAs taken in via CD36 or FATPs mediate the immunosuppressive response via eliciting Teffs exhaustion or stimulating PPAR-β and FAO in Tregs. FoxP3 also works as a critical immunosuppressive mediator by regulating FAs metabolism in Tregs. Cholesterol induces the expression of PD-1 and 2B-4 and subsequent exhaustion of Teffs to promote tumor growth. Leptin in the TME suppresses Teffs through the PD-1-STAT3-CPT1B pathway to enhance FAO and eliminate cytotoxicity. **b** FAs taken in via transporter proteins or de novo synthesis in macrophages can stimulate CPT1B and FAO, thus enhancing the secretion of immunosuppressive cytokines like ARG-1 and IL-10 or suppressing inflammatory cytokines like TNF-α, IL-6, and IL-1β. M-CSF from the TME enhances FASN expression. Macrophages with high expression of ABCG1 transport cholesterol outside and promote IL-4 expression and tumor progression. **c** MSR1 and TGFBR1 facilitate FAs transportation and LD formation in DCs, which influence antigen processing, TLR stimulation, and proliferation of DCs. **d** PUSFAs taken in by CD36 on MDSCs activate STAT3/5 and stimulate ROS production. M-CSF promotes FASN and FA production in MDSCs, which subsequently enhance immunosuppressive cytokine production, like IL-10, ARG-1, and iNOS. **e** FAs suppress the cytotoxicity of NK cells through the mTOR-PPAR signaling pathway. **f** LDs are enriched in tumor-infiltrating neutrophils due to elevated exogenous intake of FAs and downregulation of lipolysis enzyme ATGL caused by PGE2. LDs of neutrophils are then transported to tumor cells to facilitate their proliferation and progression. Oxysterol promotes the migration of neutrophils via binding to CXCR2

Due to the lack of key enzymes, CD8^+^ T cells cannot catabolize accumulated intercellular very long chain FAs or store them in LDs, resulting in severe lipotoxicity and subsequent T cell exhaustion [74]. Lipid-rich human breast cancer tissues release large quantities of FFAs to inhibit CTL-mediated tumor suppression, which can be reversed by reducing FFA levels [19] (Fig. 3a). Decreased ability to mediate FAs disables CD8^+^ T cells to sustain lipid homeostasis and compromises their anti-tumor ability. Accumulated FAs in Teffs cells enhance subsequent catabolism and FAO.

Although FAO could enhance CD8^+^ T cell anti-tumor activity in PD-1 antibody treatment [48], the opposite results are observed. It has been reported that STAT3 inhibits CD8^+^ Teffs cells by promoting FAO and reducing glycolysis. Knock-out of *Stat3* in T cells or using an FAO inhibitor enhances glycolysis and anti-tumor functions, eliminating breast tumors [75]. In this process, leptin, a secreted adipokine from adipocytes, has been reported to stimulate STAT3 and CPT1B by binding to the leptin receptor or PD-1 on CD8^+^ T cells, suppressing their glycolysis and anti-tumor ability [76] (Fig. 3a). In addition, PD-1 activation changes the metabolic program of pre-activated CD4^+^ T cells, switching from glycolysis to FAO. These mechanisms may explain the immune tolerance of the PD-1 stimulated T cells.

Tc9 cells, a subset of CD8^+^ T cells that highly express IL-9, have stronger anti-tumor ability than Tc1 cells. Microarray analysis showed that Tc9 cells had low PPAR-α/retinoid X receptor α levels, accompanied by lower expression of cholesterol synthesis enzymes (*Hmgcr* and *Sqle*), and higher expression of cholesterol efflux enzymes (*Abca* and *Abcg1*). The addition of cholesterol-derived oxysterols inhibits IL-9 expression, induces apoptosis of Tc9 cells, and results in impaired anti-tumor activity [77]. In addition to influencing cytokine secretion, cholesterol also causes exhaustion of tumor-infiltrating CD8^+^ T cells (Fig. 3a). In vitro experiments and clinical samples showed that cholesterol increased PD-1 and 2B4 expressions on CD8^+^ T cells in a dose-dependent manner, similar to the apoptosis rate [78]. However, the proliferation rate and productions of GzmB, IFN-γ, and TNF-α in CD8^+^ T cells decreased. Possible mechanisms of this phenomenon are at least partially dependent on elevated X-box binding protein 1 and endoplasmic reticulum stress [79].

### Regulator T cells need FAs to exert immunosuppressive effects

Tregs are a group of tumor-infiltrating immunosuppressive CD4^+^ T cells that mainly rely on FAO rather than glycolysis [80]. These characteristics enable them to survive in nutrient-depleted TME and exhibit immunosuppressive effects. Within the hypoxic TME, hypoxia-inducible factor-1α directs glucose away from mitochondria and promotes mitochondrial metabolism in Tregs, enhancing their ability to suppress CD8^+^ T cells [81]. Forkhead box protein P3 (FoxP3) improves FA uptake, oxidative phosphorylation, and FAO in Tregs and helps them survive in the lipotoxic tumor microenvironment to promote tumor growth and immune evasion [18, 82, 83]. Elevated CD36 and FoxP3 on Tregs strengthen lipotoxicity resistance and metabolic adaptation of Tregs in a high-lactate microenvironment by enhancing FA uptake and FAO in a PPAR-β-dependent manner [47, 82] (Fig. 3a). CD36 ablation severely decreases lipid uptake in Tregs, leading to a deceleration in tumor growth [47]. PD-1 promotes lipolysis by enhancing ATGL and upregulating CPT1A and FAO [51]. Synergic effects have been observed in combination therapy with anti-CD36 and anti-PD-1 [47]. This indicates that CD36 blockade might be a potent enhancer in current immunotherapies with reduced side effects.

After considering the metabolic signature of tumor-infiltrating Tregs, researchers considered that FA synthesis might be more important in shaping the lipid Treg pool and promoting their proliferation than FA uptake [84]. OX40, a tumor necrosis factor receptor superfamily member 4, shapes the constitution of lipids in Tregs and promotes their proliferation by enhancing FA synthesis, but not FA uptake [82]. The mouse tumor model and clinical data confirmed that the activation of sterol regulatory element-binding proteins promotes lipid synthesis and supports the local proliferation of OX40^+^ Tregs in the TME [82].

### Intercellular FAs and cholesterol exert opposite effects on TAMs and MDSCs

Macrophages and MDSCs are both derived from the bone marrow and migrate to tumor tissues. As a result, these two cell subtypes exhibit similar characteristics of lipid metabolism. Although macrophages participate in various tumoricidal immune responses, tumor-associated macrophages (TAMs) are influenced by many factors in the TME, reprogrammed to the M2 phenotype, and exert pro-tumor effects. M1 is prone to utilize glycolysis, whereas M2 mainly depends on FAO. TAMs tend to uptake more lipids via CD36 from the TME to sustain their tumor-promoting ability [85]. Moreover, TAMs can satisfy their FA demand through de novo synthesis using acetyl-CoA [86]. Macrophage colony-stimulating factor derived from Lewis lung carcinoma induced FASN expression in TAMs, stimulating tumor growth and angiogenesis in ARG1- and IL-10-dependent manner [87] (Fig. 3b). FASN inhibition significantly suppresses the expressions of TNF-α, IL-6, IL-10, and ROS in TAMs, thereby impairing their pro-tumor activity [88]. These results suggest that abnormal lipid accumulation is indispensable for TAMs to participate in the pro-tumor immune response. Alpha/beta-hydrolase domain-containing proteins (lipolytic factor of TAG) and MAGL (also named MGLL, a key lipase of monoacylglycerols) are critical enzymes in the regulation of lipid metabolic balance. Lipid accumulation in TAMs is completely restrained in MGLL-overexpressing mouse models, with elevated levels of pro-inflammatory cytokines (IL-1β, TNF-α, and IFN-γ) and decreased levels of anti-inflammatory cytokines (IL-10, ARG-1, transforming growth factor-β (TGF-β), and IL-4) [89]. TGF-β further enhances the immunosuppressive ability of TAMs by promoting LD formation through activating Mek1/2, Erk1/2, and Rsk1/2 [90]. Mek inhibition restricts LD formation and shifts the cytokine balance toward the M1 phenotype, especially rescuing the reduced ROS and NO levels [90]. It should be mentioned that the catabolism of FA is also reinforced in TAMs to generate more energy and sustain a pro-tumor phenotype. Elevated CTP1A initiates and shapes TAMs by regulating the JAK1-STAT6 axis with elevated FAO, ATP production, and oxidative stress [85]. Inhibition of FAO with etomoxir reversed the tumor-promoting effects of TAMs by decreasing their engulfment ability and downregulating IL-1β expression in a ROS-NLRP3-dependent manner [91, 92] (Fig. 3b). Moreover, receptor-interacting protein kinase 3 knockout promoted the M2 phenotype, especially PD-L1 expression in macrophages, by enhancing FAO and oxidative phosphorylation-related genes (*Cpt1a*, *Cpt1b*, *Acadvl*, and *Hadh*) [93]. These results suggest that FAs act as anti-inflammatory factors in TAMs.

MDSCs in the TME exhibit potent and comprehensive immunosuppressive effects not only by promoting tumor cell growth but also by building a complicated network with other immune cells [94, 95]. Uncontrolled lipid accumulation caused by increased lipid uptake via transport receptors (*Slc27a1*/Fatp1, *Slc27a6*/Fatp6, *Msr1*, *CD36*, and *ldlr*) accounts for the metabolic reprogramming of tumor-infiltrating MDSCs [96]. Unsaturated fatty acids (USFAs), but not SFAs, promoted intracellular LD formation and suppressed the activities of MDSCs [97]. (α-) linolenic acid (USFA) dramatically promoted the expansion and proportion of polymorphonuclear MDSCs and exerted a stronger inhibitory effect on T cells than SFAs by activating JAK-STAT3 and ROS [98] (Fig. 3d). Genetic depletion of CD36 or inhibition of STAT3/5 restricted oxidative metabolism and immunosuppressive function in MDSCs, resulting in CD8^+^ T cell-dependent tumor delay due to reduced ARG1 and iNOS [96]. Similar to TAMs, macrophage colony-stimulating factors from Lewis lung cancer cells can also elevate FASN expression in MDSCs, activating PPAR-β/δ and subsequent IL-10 secretion [87] (Fig. 3d). Moreover, cardiolipin stimulates IL-10 secretion by activating PPAR-γ in MDSCs, which reside in the lungs of tumor-bearing mice [99]. MDSCs rely more on FAO to sustain their immunosuppressive and tumor-promoting abilities [100, 101]. High CPT1 and 3-hydroxyacyl-CoA dehydrogenase expression was observed in both monocytic MDSCs and polymorphonuclear MDSCs, increasing immunosuppressive factor expression (ARG1, iNOS2, and NO) and reduced T cell proliferation rate as well as IFN production [102] (Fig. 3d). The FAO inhibitor etomoxir can reinforce the anti-tumor effects of adoptive T cell therapy and low-dose chemotherapy by inhibiting the infiltration of MDSCs [102]. This suggests that targeting FAO could limit the immunosuppressive function of MDSCs and facilitate other anti-tumor therapies.

Unlike FAs, cholesterol is an inflammatory mediator in TAMs and MDSCs. ABCG1 is highly expressed in TAMs, promoting cholesterol efflux rate and subsequent IL-4 secretion to sustain their anti-inflammatory characteristics [103] (Fig. 3b). TAMs derived from tumor tissues of Abcg1^−/−^ mice fed with a Western-like diet showed a higher apoptosis rate, shifting from the M2 phenotype to the M1 phenotype with enhanced cytotoxicity for tumor cells and reduced tumor growth. Furthermore, ABCG1 deficiency in macrophages resulted in changed intrinsic cytokine production, augmented NK cells and CD4^+^ T cell infiltration in the TME, and prevented tumor growth [104]. However, cytochrome P450 family 27 subfamily A member 1 (CYP27A1), a cytochrome p450 oxidase needed for the transformation of cholesterol to 27-hydroxycholesterol (27HC), is highly expressed on macrophages infiltrated in the breast tumor. This means that 27HC derived from macrophages is an enhancer of breast tumor growth [105]. This pro-tumor effect was compromised when CYP27A1 was inhibited. The underlying mechanism might be that 27HC or other cholesterol precursors and oxysterols polarize macrophages into the M2 phenotype by activating LXRs [106, 107].

To date, studies on cholesterol metabolism in MDSCs are limited. It has been proposed that deletion of ABCA1/G1 genes in myeloid cells blocks their cholesterol efflux and limits MDSCs and tumor growth [108]. LXR agonists stimulated cholesterol efflux and significantly decreased the proportion of MDSCs in vitro and in vivo via the LXR β nuclear hormone receptor and apolipoprotein E (ApoE) [109, 110]. In ApoE-deficient tumor-bearing mice, circulating and intra-tumor MDSCs were higher than the control and led to faster tumor growth [109]. In addition, PD-1 deletion in myeloid cells led to Erk1/2 and mammalian target of rapamycin (mTOR) C1 activation in response to granulocyte-colony stimulating factor, promoting cholesterol synthesis and differentiation of myeloid cells. This results in effective innate and adaptive antitumor responses [111].

These findings suggest that increased cholesterol content in MDSCs and TAMs might synergistically activate anti-tumor immune responses and eliminate malignant tumors. The different effects of lipids in macrophages may be partially due to the subtypes of FAs, cholesterol, and malignant tumors. As a result, further clinical applications targeting macrophage lipid metabolism should consider these contradictory results and develop more specific lipid-targeted therapies to maximize anti-tumor effects.

### High lipid content damages the antigen-presenting ability of DCs

The anti-tumor effects of DCs are mainly derived from their antigen-presenting ability. Although high lipid content renders liver DCs with greater cross-presentation ability, many other studies have demonstrated that FAs might also be immunosuppressive factors in tumor-infiltrating DCs. Diet-induced obesity caused limited expression of co-stimulatory molecules and DC-related cytokines, which induced DCs to activate T cells in tumor-bearing mice [112]. In addition, elevated lipoprotein lipase and FABP1 levels in radiation-induced thymic lymphomas lead to high serum TAG levels and subsequent DC dysfunction [113]. These results suggest that lipids are prone to mediate the pro-tumor immune response from DCs. Moreover, soluble factors within the TME limit the formation of MHC class I complexes and impair the antigen-presenting ability of DCs by upregulating their oxidized neutral lipid content [114]. SFAs and PUSFAs significantly impaired the differentiation and activation of DCs, subsequently diminishing T cell function [115]. Macrophage scavenger receptor 1 (MSR1) accounts for elevated FA levels and limited ability to prime naïve CD8^+^ T cells in DCs [116] (Fig. 3c). Blocking MSR1 impaired FA accumulation in DCs, which could stimulate the expansion and cytotoxicity of adoptively transferred tumor-specific CD8^+^ T cells in breast tumors [117]. Another lipid uptake protein, steroid receptor RNA activator 1, also affects the antigen presentation ability and immunogenicity of DCs [118]. Blocking FA uptake or impairing lipid synthesis in DCs rescued the immunosuppressive state by improving T cell-stimulating ability [113]. Besides, Zhao et.al have found that melanomas could upregulate the expression of CPT1A and strengthen FAO in DCs through Wnt5a-β-catenin-PPAR-γ signaling pathway. This FAO shift enhanced the amount and activity of indoleamine 2,3-dioxgenase-1 while suppressing the expression of IL-6 and IL-12, culminating in the generation of Treg [119].

Dysfunction of lipid metabolism in DCs results in LD accumulation. LDs impair the antigen presentation ability of DCs by decreasing MHC expression and reducing Ag-specific T cell-stimulating ability [114, 120]. Autocrine TGF-β2 in acidic TME accounts for abnormal LD accumulation in DCs, suppressing their proliferation and migratory capacity toward lymph nodes as well as CD8^+^ T cell-stimulating ability [121, 122] (Fig. 3c). SB‐431542, a potent TGF‐β I receptor inhibitor, attenuated the restraining effects of DC vaccines caused by TGF-β [123]. This research suggests that small-molecule inhibitors may regulate tumor immunity by affecting lipid metabolism.

Based on the above discussion, the exact effects of lipid accumulation within DCs remain unclear. This may be due to their organs of residence or the species of lipids. Based on their markers and morphology, DCs were divided into myeloid DCs and plasmacytoid DCs. Whether these two subtypes have distinct lipid metabolism landscapes remains unclear. Further studies should focus on detecting the specific mechanisms of lipid metabolism in tumor-associated DCs.

### Immunosuppressive effects of lipids in other immune cells

NK cells are rapid and potent responders in the anti-tumor immune response by producing cytotoxic granules, such as perforin and GzmB or inflammatory cytokines, such as IFN-γ and TNF to kill tumor cells directly [124]. Many factors in the TME cause a switch from glycolysis to oxidative phosphorylation in NK cells, which inactivates their anti-tumor properties and leads to tumor immune escape [125]. A lipid-enriched TME forced tumor-infiltrating NK cells to uptake more lipids, which inhibits the traffic of cytotoxic factors and anti-tumor functions both in vitro and in vivo due to “metabolic paralysis” [126]. Transcriptional analysis showed that high levels of mTOR stimulated PPAR-α/δ targeted genes, including the genes involved in lipid and glycerol uptake (*Cd36*, *Lpl*, and *Lrp4*), LD formation and lipases (*Lipe* and *Plin2*), and lipid metabolism (*Abca1*, *Scarb2*, and *Gyk*) in NK cells in high-fat diet mice, which displayed low cytotoxicity [127] (Fig. 3e). mTOR is a master regulator of cellular metabolism and regulates the development and activation of NK cells [128]. Knockout of CISH gene in NK cells stimulates mTOR signaling and increases NK cell metabolic fitness, which further improves their anti-tumor ability [129]. These results demonstrate that obesity influences tumor-associated NK cells in the mTOR-PPAR pathway. Therefore, PPAR-targeted therapies eliminate tumor growth by simultaneously reducing tumor cell proliferation and activating NK cell function. In addition to solid tumors, NK cells derived from individuals with diffuse large B-cell lymphoma had increased lipid metabolism and reduced IFN-γ expression [130]. Transcriptional analysis showed an enrichment of lipid metabolism-associated genes, including *Cd36*, *Fabp4*, *Fabp5*, and *Pparg*, in mice with diffuse large B cell lymphoma, which was consistent with the marked increase in neutral lipid levels. In vivo experiments also confirmed that a simulation of physiological conditions with a combination of FAs at lower concentrations resulted in significant reductions of IFN-γ and GzmB. One interesting phenomenon is that surgical treatment of tumor-bearing mice upregulates the lipid content of splenic NK cells with high CD36 and low GzmB expression [131]. This result suggests that lipid supply after malignant tumor surgery may have adverse effects on the anti-tumor immune response. In conclusion, we infer from the above discussion that cholesterol facilitates the anti-tumor ability of NK cells, while FA accumulation might not. This means that in NK cell targeting therapy, inhibiting FAs and promoting cholesterol together may result in a strengthened anti-tumor immune response.

Ns have also been found to participate in tumor-associated immune responses. Contrary to their normal abilities, such as engulfing bacteria, inducing tissue damage, and activating the immune system in infections, tumor-associated Ns are thought to participate in the pro-tumor immune response [73]. It is widely accepted that Ns facilitate lung metastasis by accelerating disseminated tumor cell migration and proliferation and awaken them by neutrophil extracellular traps [132, 133]. Ns in pre-metastatic lung tissues of the 4T1 tumor model highly express genes related to lipid absorption and LD formation with low expression of genes involved in LD degradation and β-oxidation [134]. This means that lung-resident Ns possess lipid accumulation potential, which is strengthened during cancer progression. Meanwhile, prostaglandin E2 derived from mesenchymal cells inhibits ATGL by activating *Hilpda* and facilitates LD formation in Ns. These LDs are then transported to cancer cells to promote tumor metastasis [134] (Fig. 3f). Compared with wild-type mice, *Atgl *^*−*^*/*^*−*^ mice exhibited more robust spontaneous lung metastases but unaltered primary tumor growth. Cholesterol also influences tumor-infiltrating Ns. Tumor-derived oxysterols recruit pro-tumor Ns through a CXCR2-dependent pathway and promote neoangiogenesis and immunosuppression [135]. Pertussis toxin inhibits the migration of CD11b^high^ Gr1^high^ Ly6G^+^ cells to 22(R)-hydroxycholesterol, indicating that G-protein-coupled receptors mediate the migration of these cells toward LXR ligands and subsequent activation of CXCR2 signaling [135] (Fig. 3f). Tumor-bearing mice showed better outcomes with delayed tumor growth and prolonged overall survival when blocking the oxysterol-CXCR2 axis and impairing neutrophil migration. Further research on lipid metabolism in tumor-infiltrating Ns is necessary to develop novel avenues for malignant tumor therapy, especially in limiting metastasis.

## Lipid metabolism and tumor immunotherapy

Recent studies have revealed the important roles of lipid metabolism in tumor initiation, progression, and metastasis, as well as in regulating cancer immunity. Researchers have proposed strategies targeting aberrant lipid metabolism in the tumor microenvironment. Given that a variety of inhibitors targeting lipid metabolism have been developed for the treatment of cardiovascular diseases, repurposing existing drugs to target abnormal lipid metabolism in cancers may be effective. However, it should be noted that therapies targeting lipid metabolism may influence both tumor and immune cells. To date, many treatments targeting lipid metabolism in tumors have achieved significant effects in enhancing anti-tumor immune response.

Given the fact that exogenous FA uptake largely depends on CD36 expression, the application of CD36 inhibitors in preclinical studies impedes the growth of multiple cancers and shows synergistic effects when combined with an FASN inhibitor and anti-PD-1 therapy [47]. In addition to directly eliminating tumor cells, CD36 inhibition could refine the immunosuppressive TME and suppress tumor progression by ablating the function of intra-tumor Tregs [47]. Meanwhile, blocking CD36 on DCs with monoclonal antibodies recused their antigen presentation ability by MHC class II molecules, which improved CD4^+^ T cell priming and consequently boosted anti-tumor immune response [136] (Table 2). Additionally, silencing MSR1 on DCs promoted DC vaccination efficacy [137].

In addition to FA uptake, de novo FA synthesis accounts for a large proportion of lipid sources. ACLY transforms citrate and CoA to acetyl-CoA and oxaloacetate, which links together glycolytic and lipid synthesis [138]. ACC catalyzes the transformation from acetyl-CoA to malonyl-CoA [139]. Interfering with their expression through inhibitors or siRNA inducesd tumor apoptosis, limits tumor growth [140, 141], and synergistically enhances chemotherapy or targeted drugs [142]. Adenosine 5′ monophosphate-activated protein kinase (AMPK) is a critical suppressive regulator of ACC, and metformin is a classic agonist of AMPK [143, 144]. In addition to directly influencing tumor cells [145], AMPK may also influence the lipid metabolism of tumor-associated immune cells. A phase II clinical trial reported that the application of low-dose metformin increased CD8^+^ T cell numbers and decreased tumor-associated macrophages in the TME [146] (Table 2). However, a recent study reported that depleting ACLY in macrophages did not strengthen the anti-tumor immune response in mice [147]. Stearoyl co-A desaturase (SCD) is another key enzyme in FA synthesis and is correlated with oncogenesis, tumor progression, and overall survival. SCD1 inhibitors and targeted therapies exert a synergistic suppressive effect in cancers [148]. Although SCD plays a role in inflammatory diseases, no direct evidence has revealed its relationship with tumor immunity. Acetoacetyl-CoA derived from FASN catalysis links FA biosynthesis to cholesterol biosynthesis, which promotes the formation of lipid rafts and the Toll-like receptor (TLR) 4 signaling pathway and leads to higher cytokine responses in macrophages [149]. Inhibition of lipid biosynthesis with C75, an inhibitor of FASN, in mouse bone marrow-derived macrophages reduced IL-1β, IL-10, IL-6, and TNF-α levels in response to various TLR agonists [88] (Table 2). Several next-generation compounds targeting FASN have been developed with less severe side effects and effective anti-tumor effects in preclinical studies [150–152]. Further analysis of the effects of these drugs on tumor immunity is essential.

FA decomposition is also important in tumor-associated immune cells. Specifically, inhibition of the catalytic activities of diacylglycerol acyltransferase (A922500, PF-06424439), ATGL (SML1075), and MAGL (SML0872) in macrophages attenuates their immunosuppressive capacity [153]. Therapies targeting CPT1 exert anti-tumor effects, such as diminishing the formation of invadopodia in hepatocellular carcinoma cells [154] and abrogating the pro-tumor effects of TAMs in vivo and in vivo [85] (Table 2). FAO inhibition by etomoxir had no influence on the number of F4/80^+^ macrophages in the TME, but decreased the engulfment of pancreatic ductal adenocarcinoma cells via F4/80^+^ macrophages [91]. In other types of immunosuppressive cells, etomoxir has a synergistic effect with adoptive T cell therapy and low-dose chemotherapy in inhibiting tumor-infiltrating MDSCs and promoting anti-tumor efficacy [102]. Another FAO inhibitor, perhexiline, re-sensitizes breast cancer stem cells to chemotherapy and enhances the function of CD8^+^ Teffs [76].

All these studies present solid evidence that interference in the FA metabolism in tumor-associated immune cells could recuse the immunosuppressive effects that tumor cells exert on the immune system. Once released from the metabolic block, tumor-associated immune cells switch from pro-tumor phenotype to anti-tumor phenotype and exhibit enforced tumor-eliminating ability alone or in combination with other therapies.

Except for FAs, cholesterol is also under investigation for tumor immunotherapy. LXRs are the primary sensors of dietary cholesterol and transcriptionally regulate cholesterol homeostasis by enhancing excretion and decreasing the resorption of cholesterol [155]. LXR agonists effectively induce tumor regression and prolong survival in mouse models [156]. LXR activation exhibits a pronounced anti-tumor effect in an immune-competent mouse model, indicating the participation of immune cells in LXR agonism-elicited anti-tumor activity. The LXR agonist GW3965 showed additive antitumor efficacy when combined with the frontline drugs dacarbazine and anti-CTLA-4 antibodies [110]. RGX-104 activates LXRs and suppresses the survival and immunosuppressive function of MDSCs by upregulating the expression of the pro-apoptotic Bcl-2 family member [109] (Table 2). As free cholesterol in cell membranes facilitates the immune response of T cells, inhibition of ACAT using avasimibe shows profound efficacy in reducing tumor progression with or without anti-PD-1 therapy [59] (Table 2). Co-application of avasimibe and chemotherapy also enhances the anti-tumor ability of CTL and PTX/αGC-TH-Lip in B16F10 melanoma xenograft and lung metastasis models [61] (Table 2).

**Table 2:** Strategies for lipid metabolism in tumor-infiltrating immune cells

Mechanism	Type	Example	Phase	Tumor type	Trial number	Effect	References
Fatty acids	CD36 antibody	CD36 antibody	Preclinical	Melanoma; colon cancer; lymphoma	NA	1. Ablating the function of intratumor Tregs; 2. Reducing antigen presentation ability of DC	[82, 133]
	ACC inhibitor	Metformin	Phase II	Esophageal Cancer	ChiCTR-ICR-15005940	Increasing CD8^+^ T cell amount and decreasing tumor-associated macrophages	[143]
	FASN inhibitor	C75	Preclinical	Thyroid carcinoma, neuroblastoma	NA	Reducing IL-1β, TNF-alpha, IL-6, and IL-10 levels in macrophages	[88]
	CPT1 inhibitor	Etomoxir	Preclinical	Breast, colon, lung, and prostate tumor	NA	1. Abrogating the pro-tumor effects of TAMs; 2. Inhibiting tumor-infiltrating MDSCs	[85, 102]
		perhexiline	Preclinical	Breast cancer	NA	Boosting function of CD8^+^ Teffs	[74]
Cholesterol	LXRs agonist	RGX-104	Phase I a/b	Multiple tumors	NCT02922764	Suppressing survival and immunosuppressive function of MDSCs	[109]
	ACAT inhibitor	Avasimibe	NA	Melanoma	NA	Enhancing CTL responses	[57]
Extracellular vesicles	Exosomes	(DC)-derived exosomes (DEX) loaded with the MAGE tumor antigens	Phase I	NSCLC	NA	Controlling drug release in tumors	[158]
	Liposome/lipid-NP	DCR-MYC, TKM-080301, EphA2-targeting DOPC-encapsulated siRNA	Phase I/II	Hepatoma, recurrent Solid Tumors	NCT01591356, NCT02314052, NCT02191878	Enhancing drug delivery and minimize serious side effects	[156]

In addition to their roles as an energy source or physical regulator, lipids can be remolded to effective transporters due to their physicochemical and biological properties. To date, lipid-based vesicles, including liposomes, solid lipid-based systems, lipid particles, nonionic surfactant vesicles, and micelles, have been developed as drug carriers to enhance drug delivery efficiency and have achieved promising outcomes in preclinical and clinical studies [157]. Liposomes are the first type of lipid-based nanoparticles used in cancer treatment. Liposomes have high biocompatibility and biodegradation and can deliver a variety of payloads with high target specificity, which could help chemotherapeutic drugs enter the TME and restrict serious side effects [158]. Doxil® is the first FDA-approved nano-drug encapsulating doxorubicin by liposomes, which demonstrates specific tumor localization and releases doxorubicin at the tumor site [158]. Moreover, the use of liposomes or other lipid-based nanoparticles as carriers for small interfering RNA, a promising weapon for intractable diseases, could overcome obstacles in clinical administration due to their instability in blood and negative charge density. Several clinical trials have been conducted to observe their efficiency and side effects [159].

As a result, although FA and cholesterol metabolism are important in tumor-associated immune cells, the corresponding treatments remain scarce. Given that lipid metabolism has distinct effects on different tumor-associated immune cells and even has adverse effects in the same cell type due to the types of malignant tumors and lipids, these complex phenomena and mechanisms require novel therapeutic strategies that target lipid metabolism in tumor-associated immune cells. More specifically, targeting specific lipid subtypes, enzymes, and immune cells in different malignant tumors might be a promising direction for the development of lipid-associated anti-tumor drugs.

## Conclusion and perspectives

Lipid metabolism reprogramming is now regarded as a critical characteristic of malignant tumors. In hypoxic, acidic, and nutrition-deficient TMEs, cancer and immune cells tend to rely on lipids for storage of energy, cellular building blocks for membrane formation, and sources of signaling molecules. As a result, abnormally regulated lipids in the TME can dramatically influence tumorigenesis, subsequent development, and metastasis. Meanwhile, tumor-associated immune cells residing in the TME are also affected by these ubiquitous lipid biomolecules. Tumor-infiltrating immune cells in the TME can be classified into anti-tumor and pro-tumor phenotypes based on their roles in tumor-associated immune responses. Within the TME, lipids work as a double-edged sword, which can support both anti-tumor immune response and pro-tumor immune response. For example, promoting FAO neutralizes the suppressive effects of PD-1 antibody in CD8^+^ T cells [50] and enhancing CD36 expression helps sustain effective function and long-term survival of CD8^+^ Trm cells [20]. Modification of FA metabolism in CAR-T cells refines their anti-tumor immune ability [57]. However, it has also been reported that excessive FAs hamper the anti-tumor ability of Teffs [76] and support the proliferation and immunosuppressive function of Tregs in the TME [82]. These contradictory results can also be found in other immune cells and bring us a dilemma that simply inhibits or stimulates one lipid metabolic pathway in the TME cannot obtain optimal results. Current therapies aimed at lipid metabolism in tumor-infiltrating immune cells mostly focus on single type of cell or metabolic pathways, which is far from sufficient. This means that future lipid metabolism-targeting therapies should take these contradictions into consideration to obtain a more reasonable and effective outcome in patients with malignant tumors.

In conclusion, our review summarizes numerous studies concerning lipid metabolism and relevant signaling pathways within the TME, which exert diverse and deep influences on many types of malignant tumors. However, the specific mechanisms involved remain to be elucidated. Further basic research and drug development are needed to elucidate the role of lipids in tumor immunology and to optimize existing cancer treatments.

## Data Availability

The material supporting the conclusion of this review has been included within the article.

## Abbreviations

27HC: 27-Hydroxycholesterol
ABCA/G: ATP-binding cassette transporter A/G
ACAT: Acyl coenzyme a-cholesterol acyltransferase 1/2
ACC: Acetyl-CoA carboxylase
ACLY: ATP citrate lyase
ACS: Fatty acyl CoA synthetase
AMPK: Adenosine 5′-monophosphate-activated protein kinase
ApoE: Apolipoprotein E
ARG-1: Arginase 1
ATGL: Adipose triglyceride lipase
ATP: Adenosine triphosphate
CAFs: Cancer-associated fibroblasts
CART cells: Chimeric antigen receptor T cells
CPT1: Carnitine palmitoyltransferase 1
CTL: Cytotoxic T lymphocyte
CYP27A1: Cytochrome P450 family 27 subfamily A member 1
DCs: Dendritic cells
EVs: Extracellular vesicles
FABP: Fatty acid-binding protein
FAO: Fatty acid oxidation
FASN: Fatty acid synthase
FATP: Fatty acid transport proteins
FFAs: Free fatty acids
FoxP3: Forkhead box protein P3
Glut4: Glucose transporter 4
GzmB: Granzyme B
HMGCR: 3-Hydroxy-3-methylglutaryl coenzyme A reductase
HSL: Hormone-sensitive lipase
IFN-γ: Interferon γ
IL: Interleukin
iNKT: Invariant natural killer T cells
iNOS: Inducible nitric oxide synthase
LAL: Lysosomal acid lipase
LDs: Lipid droplets
LDL: Low-density LP
LDLR: Low-density lipoprotein receptor
LPL: Lipoprotein lipase
LRs: Lipoprotein receptors
LXRs: Liver X receptors
MAGL: Monoacylglycerol lipase
M-CSF: Macrophage colony-stimulating factor
MDSCs: Myeloid-derived suppressor cells
MHC: Major histocompatibility complex
mTOR: Mammalian target of rapamycin
Ns: Neutrophils
NK cells: Natural killer cells
NKT: Natural killer T cells
NO: Nitric oxide
MSR1: Macrophage scavenger receptor 1
PD-1: Programmed death 1
PD-L1: Programmed death-ligand 1
PGE2: Prostaglandin E2
PPAR: Peroxisome proliferators-activated receptor
PUSFAs: Polyunsaturated fatty acids
ROS: Reactive oxygen species
SCD: Stearoyl co-A desaturase
SFAs: Saturated fatty acids
SREBP-1: Sterol regulatory element-binding protein 1
TAG: Triacylglyceride
TAMs: Tumor-associated macrophages
TCRs: T cell receptors
Teffs: Effector T cells
TGF-β: Transforming growth factor-β
TGFBR1: Transforming growth factor-beta receptor 1
TLR: Toll-like receptors
TME: Tumor microenvironment
TNF-α: Tumor necrosis factor-α
Treg: Regulatory T cells
Trm: Tissue-resident memory cells
